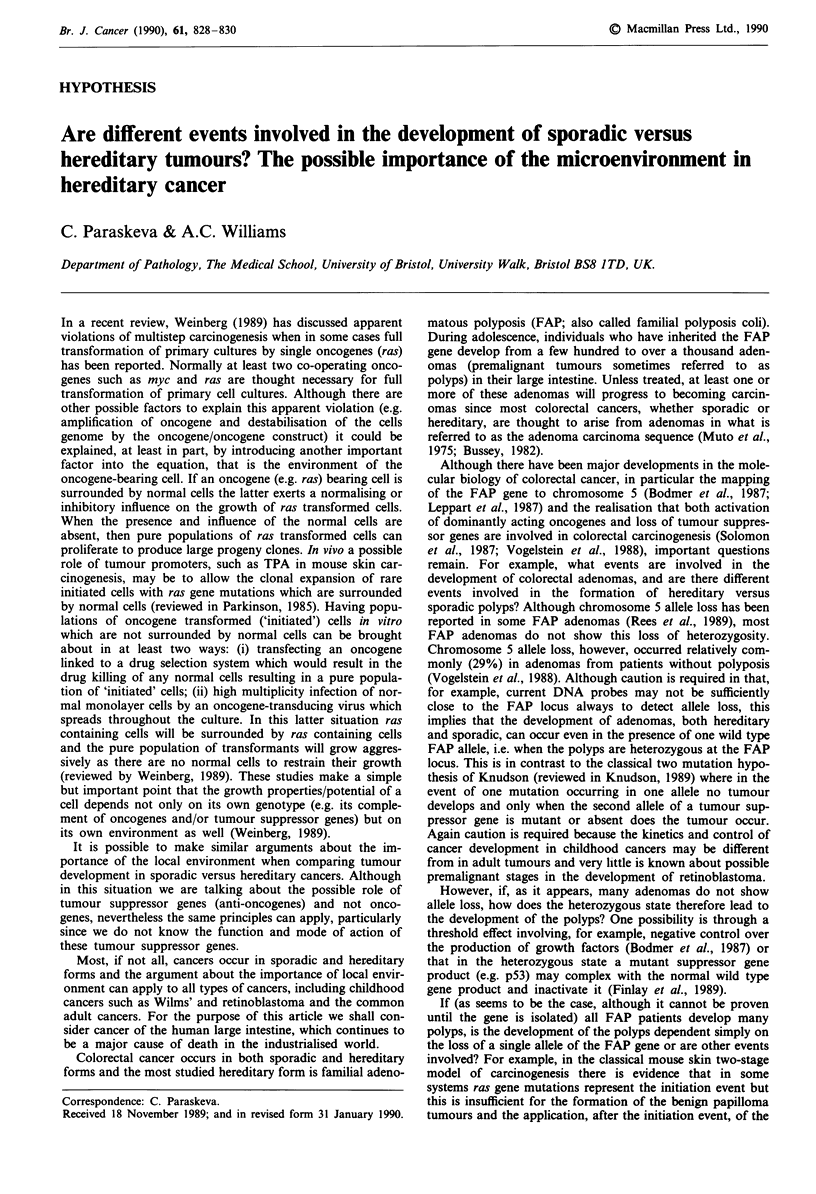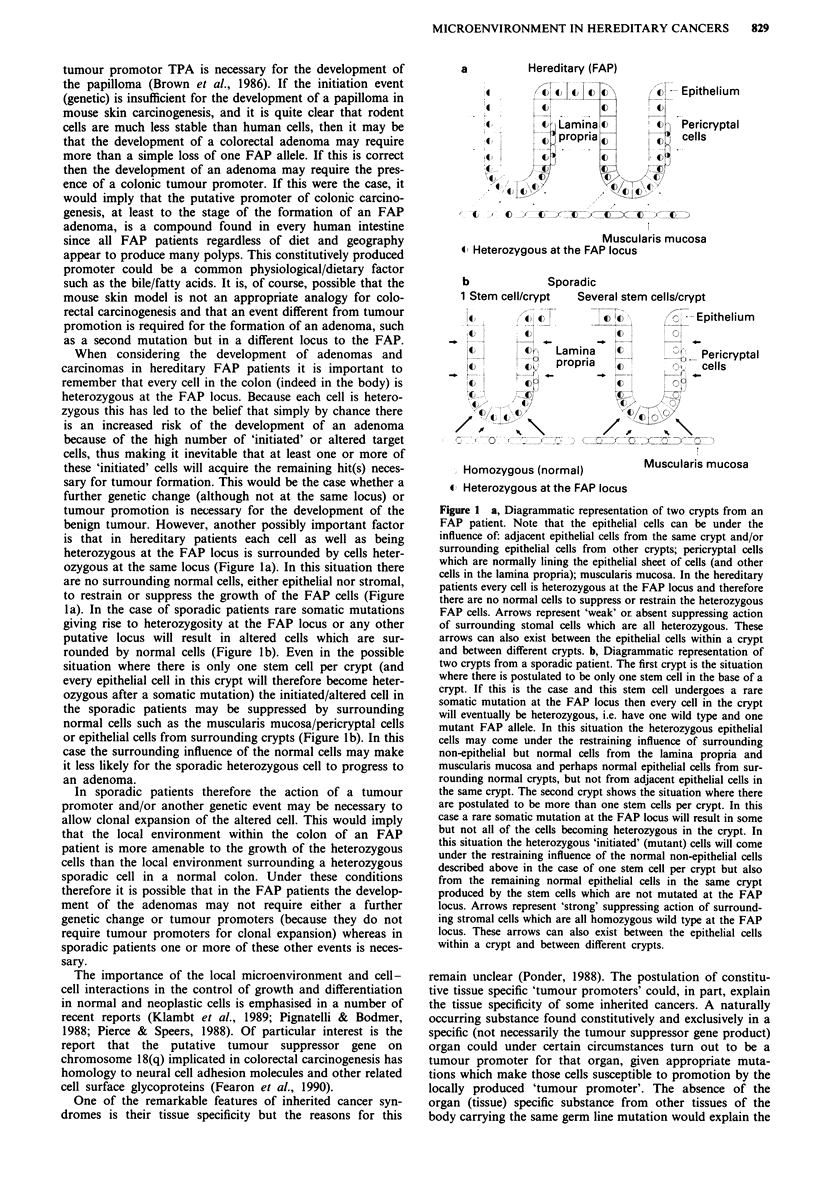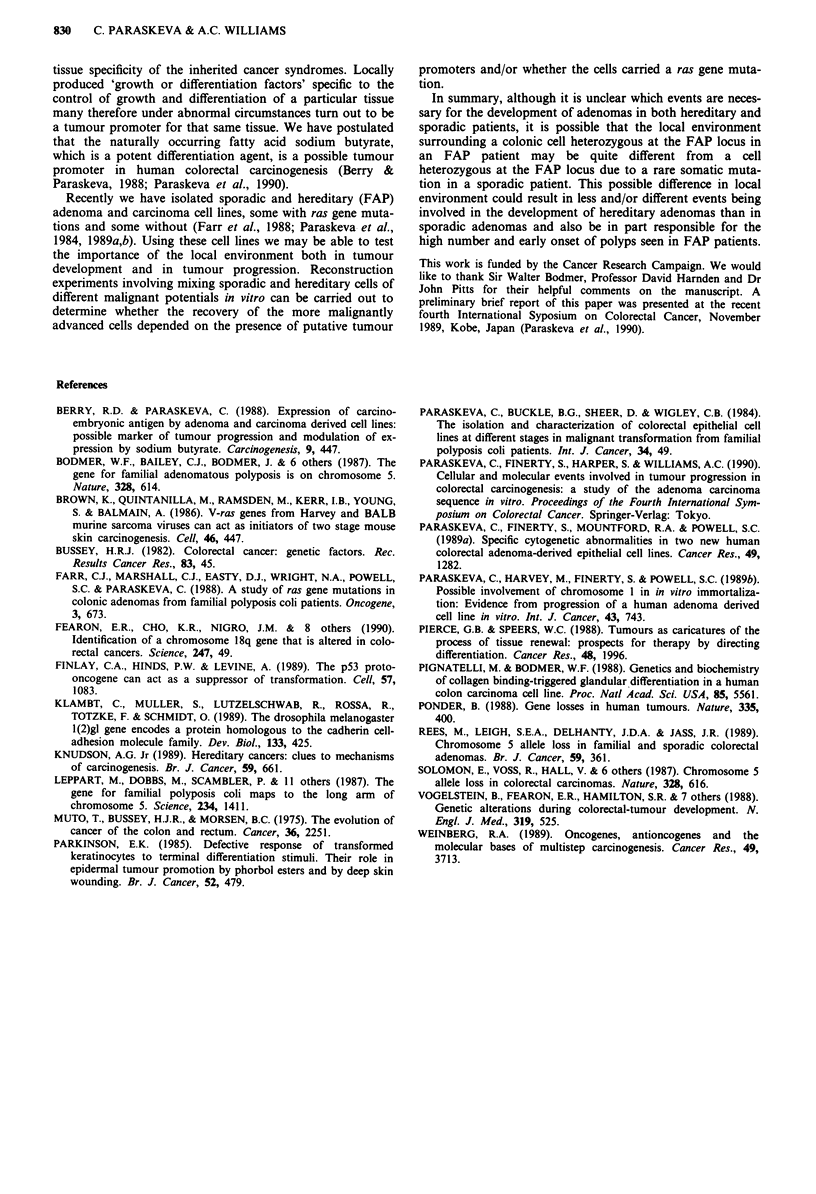# Are different events involved in the development of sporadic versus hereditary tumours? The possible importance of the microenvironment in hereditary cancer.

**DOI:** 10.1038/bjc.1990.185

**Published:** 1990-06

**Authors:** C. Paraskeva, A. C. Williams

**Affiliations:** Department of Pathology, Medical School, University of Bristol, University Walk, UK.


 © Macmillan Press Ltd., 1990

HYPOTHESIS

### Are different events involved in the development of sporadic versus hereditary tumours? The possible importance of the microenvironment in hereditary cancer

C. Paraskeva & A.C. Williams

*Department of Pathology, The Medical School, University of Bristol, University Walk, Bristol BS8 1TD, UK.*

In a recent review, Weinberg (1989) has discussed apparent violations of multistep carcinogenesis when in some cases full transformation of primary cultures by single oncogenes (*ras*) has been reported. Normally at least two co-operating oncogenes such as *myc* and *ras* are thought necessary for full transformation of primary cell cultures. Although there are other possible factors to explain this apparent violation (e.g. amplification of oncogene and destabilisation of the cells genome by the oncogene/oncogene construct) it could be explained, at least in part, by introducing another important factor into the equation, that is the environment of the oncogene-bearing cell. If an oncogene (e.g. *ras*) bearing cell is surrounded by normal cells the latter exerts a normalising or inhibitory influence on the growth of *ras* transformed cells. When the presence and influence of the normal cells are absent, then pure populations of *ras* transformed cells can proliferate to produce large progeny clones. *In vivo* a possible role of tumour promoters, such as TPA in mouse skin carcinogenesis, may be to allow the clonal expansion of rare initiated cells with *ras* gene mutations which are surrounded by normal cells (reviewed in Parkinson, 1985). Having populations of oncogene transformed ('initiated') cells *in vitro* which are not surrounded by normal cells can be brought about in at least two ways: (i) transfecting an oncogene linked to a drug selection system which would result in the drug killing of any normal cells resulting in a pure population of 'initiated' cells; (ii) high multiplicity infection of normal monolayer cells by an oncogene-transducing virus which spreads throughout the culture. In this latter situation *ras* containing cells will be surrounded by *ras* containing cells and the pure population of transformants will grow aggressively as there are no normal cells to restrain their growth (reviewed by Weinberg, 1989). These studies make a simple but important point that the growth properties/potential of a cell depends not only on its own genotype (e.g. its complement of oncogenes and/or tumour suppressor genes) but on its own environment as well (Weinberg, 1989).

It is possible to make similar arguments about the importance of the local environment when comparing tumour development in sporadic versus hereditary cancers. Although in this situation we are talking about the possible role of tumour suppressor genes (anti-oncogenes) and not oncogenes, nevertheless the same principles can apply, particularly since we do not know the function and mode of action of these tumour suppressor genes.

Most, if not all, cancers occur in sporadic and hereditary forms and the argument about the importance of local environment can apply to all types of cancers, including childhood cancers such as Wilms' and retinoblastoma and the common adult cancers. For the purpose of this article we shall consider cancer of the human large intestine, which continues to be a major cause of death in the industrialised world.

Colorectal cancer occurs in both sporadic and hereditary forms and the most studied hereditary form is familial adenomatous polyposis (FAP; also called familial polyposis coli). During adolescence, individuals who have inherited the FAP gene develop from a few hundred to over a thousand adenomas (premalignant tumours sometimes referred to as polyps) in their large intestine. Unless treated, at least one or more of these adenomas will progress to becoming carcinomas since most colorectal cancers, whether sporadic or hereditary, are thought to arise from adenomas in what is referred to as the adenoma carcinoma sequence (Muto *et al.*, 1975; Bussey, 1982).

Although there have been major developments in the molecular biology of colorectal cancer, in particular the mapping of the FAP gene to chromosome 5 (Bodmer *et al.*, 1987; Leppart *et al.*, 1987) and the realisation that both activation of dominantly acting oncogenes and loss of tumour suppressor genes are involved in colorectal carcinogenesis (Solomon *et al.*, 1987; Vogelstein *et al.*, 1988), important questions remain. For example, what events are involved in the development of colorectal adenomas, and are there different events involved in the formation of hereditary versus sporadic polyps? Although chromosome 5 allele loss has been reported in some FAP adenomas (Rees *et al.*, 1989), most FAP adenomas do not show this loss of heterozygosity. Chromosome 5 allele loss, however, occurred relatively commonly (29%) in adenomas from patients without polyposis (Vogelstein *et al.*, 1988). Although caution is required in that, for example, current DNA probes may not be sufficiently close to the FAP locus always to detect allele loss, this implies that the development of adenomas, both hereditary and sporadic, can occur even in the presence of one wild type FAP allele, i.e. when the polyps are heterozygous at the FAP locus. This is in contrast to the classical two mutation hypothesis of Knudson (reviewed in Knudson, 1989) where in the event of one mutation occurring in one allele no tumour develops and only when the second allele of a tumour suppressor gene is mutant or absent does the tumour occur. Again caution is required because the kinetics and control of cancer development in childhood cancers may be different from in adult tumours and very little is known about possible premalignant stages in the development of retinoblastoma.

However, if, as it appears, many adenomas do not show allele loss, how does the heterozygous state therefore lead to the development of the polyps? One possibility is through a threshold effect involving, for example, negative control over the production of growth factors (Bodmer *et al.*, 1987) or that in the heterozygous state a mutant suppressor gene product (e.g. p53) may complex with the normal wild type gene product and inactivate it (Finlay *et al.*, 1989).

If (as seems to be the case, although it cannot be proven until the gene is isolated) all FAP patients develop many polyps, is the development of the polyps dependent simply on the loss of a single allele of the FAP gene or are other events involved? For example, in the classical mouse skin two-stage model of carcinogenesis there is evidence that in some systems *ras* gene mutations represent the initiation event but this is insufficient for the formation of the benign papilloma tumours and the application, after the initiation event, of the

Correspondence: C. Paraskeva.
Received 18 November 1989; and in revised form 31 January 1990.

tumour promotor TPA is necessary for the development of the papilloma (Brown *et al.*, 1986). If the initiation event (genetic) is insufficient for the development of a papilloma in mouse skin carcinogenesis, and it is quite clear that rodent cells are much less stable than human cells, then it may be that the development of a colorectal adenoma may require more than a simple loss of one FAP allele. If this is correct then the development of an adenoma may require the presence of a colonic tumour promoter. If this were the case, it would imply that the putative promoter of colonic carcinogenesis, at least to the stage of the formation of an FAP adenoma, is a compound found in every human intestine since all FAP patients regardless of diet and geography appear to produce many polyps. This constitutively produced promoter could be a common physiological/dietary factor such as the bile/fatty acids. It is, of course, possible that the mouse skin model is not an appropriate analogy for colorectal carcinogenesis and that an event different from tumour promotion is required for the formation of an adenoma, such as a second mutation but in a different locus to the FAP.

When considering the development of adenomas and carcinomas in hereditary FAP patients it is important to remember that every cell in the colon (indeed in the body) is heterozygous at the FAP locus. Because each cell is heterozygous this has led to the belief that simply by chance there is an increased risk of the development of an adenoma because of the high number of 'initiated' or altered target cells, thus making it inevitable that at least one or more of these 'initiated' cells will acquire the remaining hit(s) necessary for tumour formation. This would be the case whether a further genetic change (although not at the same locus) or tumour promotion is necessary for the development of the benign tumour. However, another possibly important factor is that in hereditary patients each cell as well as being heterozygous at the FAP locus is surrounded by cells heterozygous at the same locus (Figure 1a). In this situation there are no surrounding normal cells, either epithelial nor stromal, to restrain or suppress the growth of the FAP cells (Figure 1a). In the case of sporadic patients rare somatic mutations giving rise to heterozygosity at the FAP locus or any other putative locus will result in altered cells which are surrounded by normal cells (Figure 1b). Even in the possible situation where there is only one stem cell per crypt (and every epithelial cell in this crypt will therefore become heterozygous after a somatic mutation) the initiated/altered cell in the sporadic patients may be suppressed by surrounding normal cells such as the muscularis mucosa/pericryptal cells or epithelial cells from surrounding crypts (Figure 1b). In this case the surrounding influence of the normal cells may make it less likely for the sporadic heterozygous cell to progress to an adenoma.

In sporadic patients therefore the action of a tumour promoter and/or another genetic event may be necessary to allow clonal expansion of the altered cell. This would imply that the local environment within the colon of an FAP patient is more amenable to the growth of the heterozygous cells than the local environment surrounding a heterozygous sporadic cell in a normal colon. Under these conditions therefore it is possible that in the FAP patients the development of the adenomas may not require either a further genetic change or tumour promoters (because they do not require tumour promoters for clonal expansion) whereas in sporadic patients one or more of these other events is necessary.

The importance of the local microenvironment and cell–cell interactions in the control of growth and differentiation in normal and neoplastic cells is emphasised in a number of recent reports (Klambt *et al.*, 1989; Pignatelli & Bodmer, 1988; Pierce & Speers, 1988). Of particular interest is the report that the putative tumour suppressor gene on chromosome 18(q) implicated in colorectal carcinogenesis has homology to neural cell adhesion molecules and other related cell surface glycoproteins (Fearon *et al.*, 1990).

One of the remarkable features of inherited cancer syndromes is their tissue specificity but the reasons for this remain unclear (Ponder, 1988). The postulation of constitutive tissue specific 'tumour promoters' could, in part, explain the tissue specificity of some inherited cancers. A naturally occurring substance found constitutively and exclusively in a specific (not necessarily the tumour suppressor gene product) organ could under certain circumstances turn out to be a tumour promoter for that organ, given appropriate mutations which make those cells susceptible to promotion by the locally produced 'tumour promoter'. The absence of the organ (tissue) specific substance from other tissues of the body carrying the same germ line mutation would explain the

**Figure 1** **a**, Diagrammatic representation of two crypts from an FAP patient. Note that the epithelial cells can be under the influence of: adjacent epithelial cells from the same crypt and/or surrounding epithelial cells from other crypts; pericryptal cells which are normally lining the epithelial sheet of cells (and other cells in the lamina propria); muscularis mucosa. In the hereditary patients every cell is heterozygous at the FAP locus and therefore there are no normal cells to suppress or restrain the heterozygous FAP cells. Arrows represent 'weak' or absent suppressing action of surrounding stomal cells which are all heterozygous. These arrows can also exist between the epithelial cells within a crypt and between different crypts. **b**, Diagrammatic representation of two crypts from a sporadic patient. The first crypt is the situation where there is postulated to be only one stem cell in the base of a crypt. If this is the case and this stem cell undergoes a rare somatic mutation at the FAP locus then every cell in the crypt will eventually be heterozygous, i.e. have one wild type and one mutant FAP allele. In this situation the heterozygous epithelial cells may come under the restraining influence of surrounding non-epithelial but normal cells from the lamina propria and muscularis mucosa and perhaps normal epithelial cells from surrounding normal crypts, but not from adjacent epithelial cells in the same crypt. The second crypt shows the situation where there are postulated to be more than one stem cells per crypt. In this case a rare somatic mutation at the FAP locus will result in some but not all of the cells becoming heterozygous in the crypt. In this situation the heterozygous 'initiated' (mutant) cells will come under the restraining influence of the normal non-epithelial cells described above in the case of one stem cell per crypt but also from the remaining normal epithelial cells in the same crypt produced by the stem cells which are not mutated at the FAP locus. Arrows represent 'strong' suppressing action of surrounding stromal cells which are all homozygous wild type at the FAP locus. These arrows can also exist between the epithelial cells within a crypt and between different crypts.

tissue specificity of the inherited cancer syndromes. Locally produced 'growth or differentiation factors' specific to the control of growth and differentiation of a particular tissue many therefore under abnormal circumstances turn out to be a tumour promoter for that same tissue. We have postulated that the naturally occurring fatty acid sodium butyrate, which is a potent differentiation agent, is a possible tumour promoter in human colorectal carcinogenesis (Berry & Paraskeva, 1988; Paraskeva *et al.*, 1990).

Recently we have isolated sporadic and hereditary (FAP) adenoma and carcinoma cell lines, some with *ras* gene mutations and some without (Farr *et al.*, 1988; Paraskeva *et al.*, 1984, 1989*a,b*). Using these cell lines we may be able to test the importance of the local environment both in tumour development and in tumour progression. Reconstruction experiments involving mixing sporadic and hereditary cells of different malignant potentials *in vitro* can be carried out to determine whether the recovery of the more malignantly advanced cells depended on the presence of putative tumour promoters and/or whether the cells carried a *ras* gene mutation.

In summary, although it is unclear which events are necessary for the development of adenomas in both hereditary and sporadic patients, it is possible that the local environment surrounding a colonic cell heterozygous at the FAP locus in an FAP patient may be quite different from a cell heterozygous at the FAP locus due to a rare somatic mutation in a sporadic patient. This possible difference in local environment could result in less and/or different events being involved in the development of hereditary adenomas than in sporadic adenomas and also be in part responsible for the high number and early onset of polyps seen in FAP patients.

This work is funded by the Cancer Research Campaign. We would like to thank Sir Walter Bodmer, Professor David Harnden and Dr John Pitts for their helpful comments on the manuscript. A preliminary brief report of this paper was presented at the recent fourth International Syposium on Colorectal Cancer, November 1989, Kobe, Japan (Paraskeva *et al.*, 1990).

### References

BERRY, R.D. & PARASKEVA, C. (1988). Expression of carcinoembryonic antigen by adenoma and carcinoma derived cell lines: possible marker of tumour progression and modulation of expression by sodium butyrate. *Carcinogenesis*, **9**, 447.

BODMER, W.F., BAILEY, C.J., BODMER, J. & 6 others (1987). The gene for familial adenomatous polyposis is on chromosome 5. *Nature*, **328**, 614.

BROWN, K., QUINTANILLA, M., RAMSDEN, M., KERR, I.B., YOUNG, S. & BALMAIN, A. (1986). V-*ras* genes from Harvey and BALB murine sarcoma viruses can act as initiators of two stage mouse skin carcinogenesis. *Cell*, **46**, 447.

BUSSEY, H.R.J. (1982). Colorectal cancer: genetic factors. *Rec. Results Cancer Res.*, **83**, 45.

FARR, C.J., MARSHALL, C.J., EASTY, D.J., WRIGHT, N.A., POWELL, S.C. & PARASKEVA, C. (1988). A study of *ras* gene mutations in colonic adenomas from familial polyposis coli patients. *Oncogene*, **3**, 673.

FEARON, E.R., CHO, K.R., NIGRO, J.M. & 8 others (1990). Identification of a chromosome 18q gene that is altered in colorectal cancers. *Science*, **247**, 49.

FINLAY, C.A., HINDS, P.W. & LEVINE, A. (1989). The p53 protooncogene can act as a suppressor of transformation. *Cell*, **57**, 1083.

KLAMBT, C., MULLER, S., LUTZELSCHWAB, R., ROSSA, R., TOTZKE, F. & SCHMIDT, O. (1989). The drosophila melanogaster 1(2)gl gene encodes a protein homologous to the cadherin cell-adhesion molecule family. *Dev. Biol.*, **133**, 425.

KNUDSON, A.G. Jr (1989). Hereditary cancers: clues to mechanisms of carcinogenesis. *Br. J. Cancer*, **59**, 661.

LEPPART, M., DOBBS, M., SCAMBLER, P. & 11 others (1987). The gene for familial polyposis coli maps to the long arm of chromosome 5. *Science*, **234**, 1411.

MUTO, T., BUSSEY, H.J.R., & MORSEN, B.C. (1975). The evolution of cancer of the colon and rectum. *Cancer*, **36**, 2251.

PARKINSON, E.K. (1985). Defective response of transformed keratinocytes to terminal differentiation stimuli. Their role in epidermal tumour promotion by phorbol esters and by deep skin wounding. *Br. J. Cancer*, **52**, 479.

PARASKEVA, C., BUCKLE, B.G., SHEER, D. & WIGLEY, C.B. (1984). The isolation and characterization of colorectal epithelial cell lines at different stages in malignant transformation from familial polyposis coli patients. *Int. J. Cancer*, **34**, 49.

PARASKEVA, C., FINERTY, S., HARPER, S. & WILLIAMS, A.C. (1990). Cellular and molecular events involved in tumour progression in colorectal carcinogenesis: a study of the adenoma carcinoma sequence *in vitro*. *Proceedings of the Fourth International Symposium on Colorectal Cancer*. Springer-Verlag: Tokyo.

PARASKEVA, C., FINERTY, S., MOUNTFORD, R.A. & POWELL, S.C. (1989*a*). Specific cytogenetic abnormalities in two new human colorectal adenoma-derived epithelial cell lines. *Cancer Res.*, **49**, 1282.

PARASKEVA, C., HARVEY, M., FINERTY, S. & POWELL, S.C. (1989*b*). Possible involvement of chromosome 1 in *in vitro* immortalization: Evidence from progression of a human adenoma derived cell line *in vitro*. *Int. J. Cancer*, **43**, 743.

PIERCE, G.B. & SPEERS, W.C. (1988). Tumours as caricatures of the process of tissue renewal: prospects for therapy by directing differentiation. *Cancer Res.*, **48**, 1996.

PIGNATELLI, M. & BODMER, W.F. (1988). Genetics and biochemistry of collagen binding-triggered glandular differentiation in a human colon carcinoma cell line. *Proc. Natl Acad. Sci. USA*, **85**, 5561.

PONDER, B. (1988). Gene losses in human tumours. *Nature*, **335**, 400.

REES, M., LEIGH, S.E.A., DELHANTY, J.D.A. & JASS, J.R. (1989). Chromosome 5 allele loss in familial and sporadic colorectal adenomas. *Br. J. Cancer*, **59**, 361.

SOLOMON, E., VOSS, R., HALL, V. & 6 others (1987). Chromosome 5 allele loss in colorectal carcinomas. *Nature*, **328**, 616.

VOGELSTEIN, B., FEARON, E.R., HAMILTON, S.R. & 7 others (1988). Genetic alterations during colorectal-tumour development. *N. Engl. J. Med.*, **319**, 525.

WEINBERG, R.A. (1989). Oncogenes, antioncogenes and the molecular bases of multistep carcinogenesis. *Cancer Res.*, **49**, 3713.